# Biopolymers as Seed-Coating Agent to Enhance Microbially Induced Tolerance of Barley to Phytopathogens

**DOI:** 10.3390/polym16030376

**Published:** 2024-01-30

**Authors:** Aizhamal Usmanova, Yelena Brazhnikova, Anel Omirbekova, Aida Kistaubayeva, Irina Savitskaya, Lyudmila Ignatova

**Affiliations:** 1 Faculty of Biology and Biotechnology, Al-Farabi Kazakh National University, Almaty 050038, Kazakhstan; aizhamalduszhanovna@mail.ru (A.U.);; 2 Scientific Research Institute of Biology and Biotechnology Problems, Al-Farabi Kazakh National University, Almaty 050038, Kazakhstan

**Keywords:** polyhydroxyalkanoate, polysaccharides, dynamic viscosity, seed coating, beneficial microorganisms, biocontrol

## Abstract

Infections of agricultural crops caused by pathogen ic fungi are among the most widespread and harmful, as they not only reduce the quantity of the harvest but also significantly deteriorate its quality. This study aims to develop unique seed-coating formulations incorporating biopolymers (polyhydroxyalkanoate and pullulan) and beneficial microorganisms for plant protection against phytopathogens. A microbial association of biocompatible endophytic bacteria has been created, including *Pseudomonas flavescens* D5, *Bacillus aerophilus* A2, *Serratia proteamaculans* B5, and *Pseudomonas putida* D7. These strains exhibited agronomically valuable properties: synthesis of the phytohormone IAA (from 45.2 to 69.2 µg mL^−1^), antagonistic activity against *Fusarium oxysporum* and *Fusarium solani* (growth inhibition zones from 1.8 to 3.0 cm), halotolerance (5–15% NaCl), and PHA production (2.77–4.54 g L^−1^). A pullulan synthesized by *Aureobasidium pullulans* C7 showed a low viscosity rate (from 395 Pa·s to 598 Pa·s) depending on the concentration of polysaccharide solutions. Therefore, at 8.0%, *w*/*v* concentration, viscosity virtually remained unchanged with increasing shear rate, indicating that it exhibits Newtonian flow behavior. The effectiveness of various antifungal seed coating formulations has been demonstrated to enhance the tolerance of barley plants to phytopathogens.

## 1. Introduction

According to the estimates of the Food and Agriculture Organization (FAO), 20–40% of global crop losses are related to plant diseases, with 42% of those attributed to infections caused by pathogenic fungi. *Fusarium* fungi are common pathogens of cereals, including barley, and can cause diseases such as Fusarium head blight, seedling blight, root rot, and Fusarium crown rot throughout their life cycle. In addition, many species of *Fusarium* are capable of producing mycotoxins (deoxynivalenol, nivalenol, HT2/T2, zearalenone), even in some cases in the absence of severe disease symptoms [1].

The use of microorganisms and their metabolites as bio-control agents is one of the most promising methods for the effective and safe protection of plants. The widespread use of antibiotics in the food industry, agriculture, and medicine leads to an increase in antibiotic resistance of pathogenic microorganisms. In this regard, endophytes have advantages over other biocontrol agents, as they are producers of many biologically active metabolites, such as phenolic acids, alkaloids, quinones, steroids, saponins, tannins, and terpenoids. Microbiological strategies for protecting agricultural crops are based on the plant growth-promoting properties of these strains.

Biopolymers can also be used in the development of plant protection products against phytopathogens. They are non-toxic and biodegradable and can be obtained from renewable sources, making them suitable for use in organic farming. Additionally, they can interact with many hydrophobic and hydrophilic compounds in more complex formulations. Biopolymers play a protective role for plants against pathogenic fungi through several mechanisms [2]. Polymers can directly interact with fungi, suppressing spore germination and mycelium growth, as demonstrated, for example, with chitosan [3,4]. They can act as effective elicitors, inducing the plant immune system to fight pathogens [5]. They can also be used as carriers for active ingredients with controlled release [2].

Among biopolymers, the most used are carboxymethyl cellulose, chitosan, xanthan gum, gum arabic, polyvinyl alcohol, starch, gelatin, polyacrylamide, and alginates. These polymers are used for treating the seeds of turnips, tomatoes, chickpeas, corn, beans, eggplants, okra, chili peppers, guar, pumpkins, cucumbers, lupine, clover, soybeans, and wheat [6,7,8].

One of the promising microbial polymers for seed coating is pullulan. It is a water-soluble, low-viscosity polysaccharide that has the property of biodegrading under the action of microorganisms. Pullulan has oxygen barrier properties, excellent moisture retention, and also prevents the growth of pathogens. Additionally, pullulan is a prebiotic—a substance that stimulates the growth and development of microorganisms [9,10].

Polyhydroxyalkanoates (PHAs) are of great interest as well, as they are non-toxic, biodegradable, and biocompatible polymers [11]. Previous studies have reported the use of PHA with the addition of polycaprolactone to obtain biodegradable films for rice seed germination [12]. Furthermore, some PHAs exhibit antagonistic activity against bacteria [13,14]. In our previous studies, we demonstrated that PHA produced by the strain *Pseudomonas fluorescens* D5 has pronounced antifungal activity against *Fusarium graminearum*, *Fusarium solani*, *Fusarium oxysporum* [15], and *Penicillium expansum* [16].

However, there is no information about the use of a mixture of PHA with pullulan in the composition of seed coatings. Therefore, this work is aimed at developing unique compositions for seed treatment, including effective microorganisms and biopolymers (PHA and pullulan) as seed coating agents to enhance the microbially induced tolerance of plants to phytopathogenic fungi.

The main objectives of the present study are as follows: (1) formation of a microbial association with agronomically valuable properties, (2) investigation of the rheological properties of polysaccharide solutions, and (3) study of various seed coating types for barley tolerance to *Fusarium*.

This study is significant for a better understanding of the effect of seed coating on the microbially induced tolerance of barley to phytopathogens. In this research, a new opportunity is proposed for the use of pullulan as a seed coating agent, expanding the areas of application for microbial polymers. The excellent gelling and thickening properties, as well as the biodegradability and non-toxicity of the investigated biopolymers, pullulan, and PHA, make them promising for use in antifungal formulations for seed treatments.

## 2. Materials and Methods

The following strains were used in the present study:

*Bacillus aerophilus* A2 (accession number OQ569360) isolated from leaves of peppermint (*Mentha piperita*);
*Pseudomonas flavescens* D5 (accession number OP642636) isolated from flowers of common chicory (*Cichórium intybus*);
*Serratia proteamaculans* B5 (accession number OR858823) isolated from the leaves of Iris;
*Bacillus simplex* B9 (accession number OR864231) isolated from the roots of wormwood (*Artemisia absinthium*);
Pseudomonas putida D7 (accession number OR863903) isolated from the roots of Echinacea (*Echinacea purpurea*);
*Aureobasidium pullulans* C7 (accession number OR864236) isolated from dark chestnut soil;
*Bacillus thuringiensis* C8 (accession number OR858828) isolated from the surface of apples.

### 2.1. Production of IAA

To determine the amount of indole-3-acetic acid (IAA) produced by microorganisms, a colorimetric method was employed. Isolates were cultivated in nutrient broth for 48 h at 28 °C. After incubation, the culture was centrifuged at 6000× *g* for 20 min. The supernatant, with a volume of 1 mL, was mixed with 2 mL of Salkowski reagent. The optical density was measured at 530 nm. The concentration of IAA was expressed in μg mL^−1^ [17].

### 2.2. The Antifungal Properties of Microorganisms

The antifungal activity was determined using the agar disk diffusion method. Bacteria were cultured for 48 h in nutrient broth at 28 °C with aeration. Bacterial cultures were spread on the surface of nutrient agar as a continuous lawn in a volume of 100 µL, and after 48 h of growth, 5 mm diameter disks were cut. Disks with bacterial culture were placed on Petri dishes previously inoculated with a continuous lawn of phytopathogenic test cultures (*Fusarium solani*, *Fusarium oxysporum*) at a concentration of 10^6^ spores mL^−1^. A nutrient agar disk served as a control. The Petri dishes were incubated at 28 °C for 72 h. The antifungal activity was assessed by measuring the diameter of the growth inhibition zone of the tested phytopathogens [18].

### 2.3. Determination of Microbial Halotolerance

To assess the halotolerance of bacteria, nutrient agar medium supplemented with NaCl at concentrations of 5%, 10%, 15%, and 25% was used. Microorganisms were inoculated using the streak method. Strains capable of cultivation at different salt concentrations were selected based on the research results.

### 2.4. PHA Production Assay

Strains producing PHA were cultivated in liquid MSM medium at 28 °C for 48 h at 150 rpm. The medium composition (g·L^−1^) was as follows: MgSO_4_·7H_2_O—0.1; KH_2_PO_4_—0.68; K_2_HPO_4_—1.73; NaCl—4.0; NH_4_NO_3_—1.0; FeSO_4_·7H_2_O—0.03; CaCl_2_·2H_2_O—0.02; and glucose—5.0 [19]. Subsequently, the suspension was centrifuged at 6000× *g* for 10 min, the supernatant was decanted, and PHA was extracted from the residue. Sodium hypochlorite and hot chloroform were added to the residue at a 1:1 ratio, and the mixture was kept at 30 °C for 1 h. The suspension was then centrifuged at 6000× *g* for 15 min, and the upper and middle layers were removed. The residue was precipitated with a 1:1 mixture of ethanol and acetone, dried at 35 °C, and weighed [20].

### 2.5. Microbe–Microbe In Vitro Compatibility Test

Five bacterial strains (*Pseudomonas flavescens* D5, *Bacillus aerophilus* A2, *Serratia myotis* B5, *Bacillus simplex* B9, and *Pseudomonas putida* D7) were used for in vitro compatibility test. The agar diffusion method was selected to determine biocompatibility. Bacterial strains were separately grown on nutrient agar at 28 °C for 24 h. Then, colonies were transferred to nutrient broth and incubated overnight at 28 °C at 160 rpm [21].

A volume of 100 µL of the test microorganism (0.5 McFarland) was spread on the surface of nutrient agar. Sterile filter paper disks (d = 5 mm) were inoculated with the overnight bacterial culture adjusted to a concentration of 0.5 McFarland. Inoculated disks were placed on Petri dishes (4 disks per each) with the test microorganism, and each was incubated in darkness at 28 °C for 4 days. Experiments were conducted with three replicates.

### 2.6. Extraction of Polysaccharide

For the extraction of polysaccharides, the 4-day-old culture of *A pullulans* C7 and 3-day-old culture of *B. thuringiensis* C8 were centrifuged for 15 min at 10,000× *g*. The fungal polysaccharide was precipitated with a double volume of 96% ethanol and the bacterial polysaccharide with a triple volume of alcohol. The yield coefficient for biomass (P/X) was calculated as a ratio of production of EPS to the dry biomass and expressed in percent. The yield coefficient for substrate (P/S) was calculated as a ratio of production of EPS to the utilized glucose and expressed in percent [22].

### 2.7. Measurement of Dynamic Viscosity

The dynamic viscosity of the polymer’s solution with different concentrations—2, 4, 6, 8, 10, and 12% *w*/*v*—was performed using a rotational Ametek Brookfield DVPlus viscometer with a ULA spindle, at different shear rates, ranging from 0.1 to 500 s^−1^ at 25 °C [23]. The viscosimetric analyses of the samples were performed at 25 °C.

### 2.8. Development of Various Options for Processing Barley Seeds

Various antifungal formulations for seed treatments were developed, including (1) bacterial strain suspension, (2) polymer mixture, and (3) bacterial strain suspension + polymer mixture.

Bacterial strains were separately cultured in nutrient broth for 48 h at 180 rpm and 28 °C. The cultures were then centrifuged at 6000× *g* for 10 min and resuspended in a phosphate-buffered saline (PBS (g L^−1^), 8.0 NaCl, 0.2 KCl, 1.44 Na_2_HPO_4_, and 0.24 KH_2_PO_4_). The optical density of each bacterial suspension was adjusted to 10^8^ CFU mL^−1^ Strain suspensions were mixed in equal proportions.

The polymer mixture was prepared using PHA at a concentration of 0.05% and pullulan at a concentration of 2% (wt./vol.), incorporated into phosphate-buffered saline.

For coating of seeds simultaneously in a bacterial suspension and a polymer blend, PHA at a concentration of 0.05% and pullulan at a concentration of 2% (wt./vol.) were introduced into a mixture of bacterial suspensions.

### 2.9. Pot Experiments

To conduct the research, barley seeds sterilized in a 5% sodium hypochlorite solution were used. Subsequently, the seeds were rinsed with sterile water and sown on nutrient agar medium [22] to ensure the absence of bacteria on the seed surface.

Phytopathogenic load conditions were simulated by introducing a suspension of the phytopathogenic fungus *F. oxysporum* into the soil at a titer of 10^8^ spores mL^−1^, with 2 mL of the suspension per 100 g of soil.

Experiment options:

T1—Untreated seeds;
T2—Untreated seeds + *Fusarium oxysporum;*
T3—Seed treatment with bacterial suspension + *Fusarium oxysporum;*
T4—Seed coating with polymeric mixture + *Fusarium oxysporum;*
T5—Simultaneous seed coating with bacterial suspension and polymeric mixture of *Fusarium oxysporum.*

Pre-sterilized seeds were immersed in various antifungal formulations, followed by transferring the seeds to 0.1 M CaCl_2_. After coating, the seeds were dried for 20 min before planting.

In each pot containing 300 g of sterile soil, 10 barley seeds were planted. The experiment was conducted under sterile conditions with three replicates. The plants were grown for 12 days.

### 2.10. Determination of Free Proline Concentration

The content of free proline was determined using a non-heated acidic ninhydrin reagent prepared as follows: (1.25 g ninhydrin + 30 mL glacial acetic acid + 20 mL 6 M H_3_PO_4_). A portion of fresh plant tissue from a leaf blade (200 mg) was homogenized in 10 mL of a 3% aqueous solution of sulfosalicylic acid and left for 1 h in a water bath at 100 °C.

Subsequently, 1.5 mL of glacial acetic acid, 1.5 mL of ninhydrin reagent, and 1.5 mL of the prepared extract were poured into a clean test tube. The samples were incubated for 1 h in a water bath at 100 °C and then rapidly cooled to room temperature. After artificial cooling (using cold water or ice), the optical density of the reaction products was measured at a wavelength of 520 nm using a spectrophotometer. Proline content values were calculated using a calibration curve, constructed using chemically pure proline [24].

### 2.11. Determination of Chlorophyll Concentration

To obtain an ethanolic extract, 2 g of leaves were sliced and thoroughly ground in a mortar, gradually adding 96% ethanol in small portions (a total of 10 mL). The extract was centrifuged for 15 min at 6000× *g* [25]. Photocolorimetry was carried out using a spectrophotometer at wavelengths of 665 and 649 nm in a cuvette with an optical path length of 1 cm. The comparison cuvette was filled with 96% ethanol. The pigment concentration was determined using the following formula:

$${Chl}_{a}=13.95\times A_{665}-6.88\times A_{649}$$

$${Chl}_{b}=24.96\times A_{649}-7.32\times A_{665}$$

### 2.12. Preparation of the Extract for the Determination of Antioxidant Enzymes

Antioxidant enzyme activity was determined spectrophotometrically based on the rate of NADH oxidation using the method [26]. For this purpose, plant material (1.5–2 g) was homogenized with an extracting medium containing 50 mM K-phosphate buffer (pH 7.5), 1 mM EDTA, 0.3%, 1 mM ascorbic acid, filtered and centrifuged (15 min, 8000× *g*). The obtained supernatant was used to determine the activity of the enzymes.

#### 2.12.1. Investigation of Catalase Activity

Catalase activity was determined using H_2_O_2_ according to the method [26]. The reaction mixture consisted of 15 mM H_2_O_2_, 100 mM K-phosphate buffer (pH 7.0), and 0.1 mL of the sample. Changes in optical density were measured at 240 nm, and activity was calculated using the extinction coefficient ε = 0.03 mM^−1^ cm^−1^. All experiments were conducted in triplicate and expressed in units per milligram of protein.

#### 2.12.2. Investigation of Ascorbate Peroxidase Activity

The activity of ascorbate peroxidase was determined in a medium with the following composition: 50 mM K-phosphate buffer pH 7.0, 0.5 mM ascorbate, and 0.2 mM H_2_O_2_. The reaction was initiated by adding 0.1 mL of the sample [27]. Changes in optical density were measured at 290 nm. Enzyme activity was calculated using the extinction coefficient ε = 2.8 mM^−1^ cm^−1^ and expressed as 1 mmol of ascorbate min^−1^ per mg protein.

#### 2.12.3. Investigation of Guaiacol Peroxidase Activity

Guaiacol peroxidase activity was determined using a spectrophotometric method, considering absorption due to guaiacol oxidation [28]. The reaction mixture consisted of 50 mM phosphate buffer (pH 7), 9 mM guaiacol, 10 mM H_2_O_2_, and 0.2 mL of the sample. Optical density was measured at 470 nm for 1 min, and enzyme activity was calculated using the extinction coefficient ε = 26.6 mM^−1^ cm^−1^, expressed as 1 mmol of ascorbate min^−1^ per mg protein.

### 2.13. Statistical Analysis

All the data are presented as the mean ± standard deviation (SD) of three replicates. The data were processed by the standard methods of one-way analysis of variance (ANOVA) using the software Statistica version 10.0 (TIBCO Software Inc., Palo Alto, CA, USA). Tukey’s honestly significant difference (HSD) test (*p* < 0.05) was performed for multiple comparisons to estimate significant differences between means.

## 3. Results

### 3.1. Characterization of the Biological Activity of Endophytic Bacteria

For the application of microorganisms both to enhance plant growth and to protect them from adverse factors, a crucial step is the selection of strains possessing a set of beneficial properties. In the first stage of the research, the agronomically valuable properties of five endophytic bacterial strains were investigated (Table 1).

One of the well-known mechanisms for improving and regulating plant growth by microorganisms is their ability to synthesize various phytohormones. The stimulation of plant growth resulting from the application of microorganisms is predominantly associated with their ability to synthesize auxins, primarily IAA [29]. All examined bacteria demonstrated the ability to produce IAA (Table 1), except for the *Bacillus simplex* B9 strain. The highest concentration of IAA was found in the *Pseudomonas putida* D7 strain (Table 1). The amount of produced IAA varied between 45.2 and 69.2 μg mL^−1^ depending on the strain, which is similar to or significantly higher than that observed in other endophytic bacterial strains [30,31].

The next criterion for assessing the biological activity of the strains was the evaluation of their resistance to adverse environmental factors.

Among the adverse factors of biotic nature, phytopathogenic microflora plays a key role. Infections of agricultural crops caused by pathogenic fungi are among the most widespread and harmful, as they not only reduce the quantity of the harvest but also significantly degrade its quality due to the accumulation of mycotoxins [32]. One of the positive effects of bacteria on crops is their ability to protect plants from phytopathogens through direct and indirect mechanisms [33].

**Figure 1:**
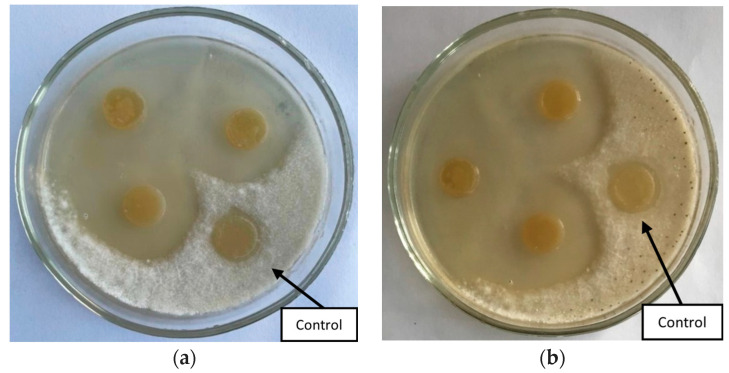
Inhibition of the growth of the phytopathogenic fungus *Fusarium solani* by strains (**a**) *Bacillus simplex* B9 and (**b**) *Serratia proteamaculans* B5.

The study of the antagonistic activity of bacterial strains against *Fusarium solani* and *Fusarium oxysporum* showed that three out of five strains inhibit the growth of phytopathogens (Figure 1). The zones of growth suppression ranged from 1.8 to 3.0 cm (Table 1).

Salinization of soils is one of the most crucial abiotic stress factors that negatively impact plant privity [34]. The application of salt-tolerant growth-promoting bacteria may contribute to stress alleviation and enhance the resilience of crops grown in saline soils [34].

**Figure 2:**
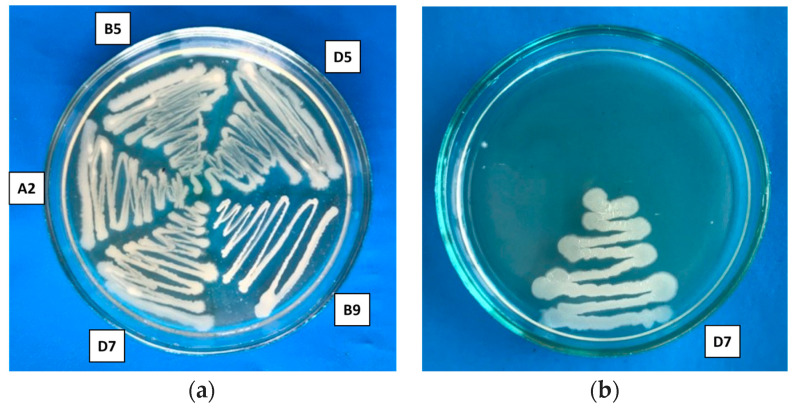
Growth of bacterial strains on medium containing (**a**) 5% NaCl and (**b**) 15% NaCl. D5—*Pseudomonas flavescens* D5, A2—*Bacillus aerophillus* A2, B5—*Serratia proteamaculans* B5, B9—*Bacillus simplex* B9, D7—*Pseudomonas putida* D7.

In the study of halotolerance, it was shown that all strains were resistant to a salt concentration of 5%, and one strain, *Pseudomonas putida* D7, demonstrated the ability to grow in a medium with 15% NaCl (Table 1, Figure 2). According to the classification, the investigated strains are moderately halophilic, exhibiting optimal growth at NaCl concentrations ranging from 3% to 15% (~0.5–2.7 M). Halophilic bacteria have several advantages compared to other microorganisms, as they possess high metabolic activity, allowing them to grow in extreme conditions and produce a variety of valuable biologically active compounds, including those with antimicrobial properties [35].

PHA is a class of polyesters of various hydroxyalkanoic acids, which are synthesized by many Gram-positive and Gram-negative bacteria and accumulate intracellularly [33]. In the present study, the strains *Ps. flavescens* D5 and *B. aerophillus* A2 demonstrated the ability to produce PHA (Table 1).

### 3.2. Biocompatibility Assessment of Strains

Currently, the advantages of preparations based on microbial consortia over monocultures are convincingly confirmed, as the biotechnological potential of microorganisms in such preparations is more fully realized. There are several advantages of multi-component preparations: multiplicity of action, synergistic effect, increased stability and adaptability to different agro-climatic conditions, the ability to utilize inhomogeneous substrates in composition, and more complete utilization of the functional capabilities of microorganisms [36,37,38].

In the development of multi-strain inoculants, it is crucial to consider the type of relationships between microorganisms and the possibility of their combination. Therefore, the next stage of the research was the in vitro testing of the selected strains for compatibility during their co-cultivation on a solid nutrient medium (Table 2).

It was shown that during co-cultivation, four out of five strains did not suppress the growth and development of each other (Table 2), indicating their compatibility and the possibility of including them in the composition of a multi-strain inoculant. The identified biocompatibility of the studied strains indicates the absence of competition between them and insensitivity to the produced extracellular metabolites with antagonistic properties. An exception was the *B.simplex* B9 strain, which demonstrated pronounced incompatibility with most of the investigated bacterial strains (Table 2). Thus, for seed treatment in subsequent experiments, four out of five strains that showed compatibility were used.

### 3.3. Biosynthesis of Microbial Exopolysaccharides and Their Rheological Properties

In addition to plant-beneficial microorganisms, such ingredients of seed coating as binders that help to release a suitable amount of plant-beneficial microorganisms in physiologic conditions and ensure the adherence and cohesion of the material on the seed surface and keep the ingredients active are used [37,39,40]. The microbial polymer solution should be water-soluble with a low viscosity for complete atomization of the liquid onto seeds [40].

Earlier, we isolated strains *Aureobasidium pullulans* C7 [41] and *Bacillus thuringiensis* C8, which showed the ability to biosynthesize exopolysaccharide (EPS). The *A. pullulans* C7 strain synthesized 12.53 ± 0.48 g L^−1^ exoglycan on the 4th day of fermentation, and the yield coefficient for biomass was 349.02% (Table 3). This indicates that in this medium the substrate is utilized to a greater extent for the formation of EPS than for the formation of cell mass. The amount of polysaccharide accumulated by the studied strain is comparable with the data of other researchers [42,43].

The strain *B. thuringiensis* C8 produced 3.97 g L^−1^ of exoglycan (Table 3). The yield coefficient for bacterial biomass indicates the potential of this strain as a producer of EPS. The ability of strains of the genus *Bacillus*, including *B. thuringiensis*, to produce EPS is confirmed in the works of other researchers [44,45].

Further, measurements of dynamic viscosity were made for polymer solutions obtained by cultivation of *A. pullulans* C7 and *B.thuringiensis* C8.

**Figure 3:**
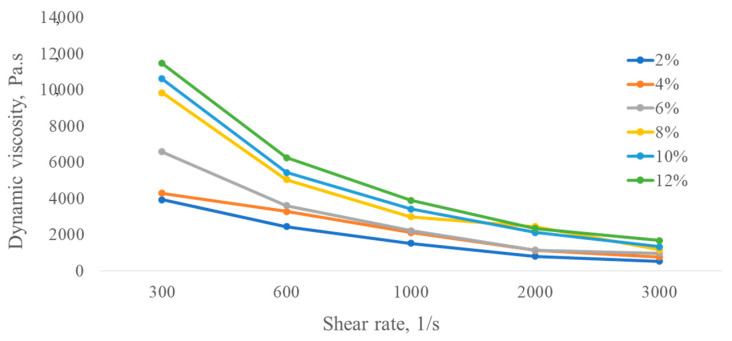
Viscosity of exopolysaccharide solutions produced by *B. thuringiensis* C8 in different concentrations.

The dynamic viscosity of each concentration solution produced by *B. thuringiensis* C8 obviously decreased with the increase in shear rate (Figure 3), showing a shear-thinning behavior, which means that this kind of polysaccharide solution belongs to non-Newtonian fluid (or pseudoplastic flow behavior). Solutions of polymers in water and at the same concentrations can sometimes have oppositional behaviors, i.e., Newtonian or non-Newtonian fluids, depending on their structures, molecular weights, and polymer microbial producers [46,47].

It was also noted that the viscosity increased with increasing polymer solution concentration; however, at lower concentrations, the rheological measurements became erratic. The shear-thinning phenomenon could be due to the rate of formation of new entanglements lower than the externally imposed disruption rate with an increase in shear rate. Another distinguishing feature is that the microbial solution showed comparatively high viscosity rates at all dilute concentrations.

**Figure 4:**
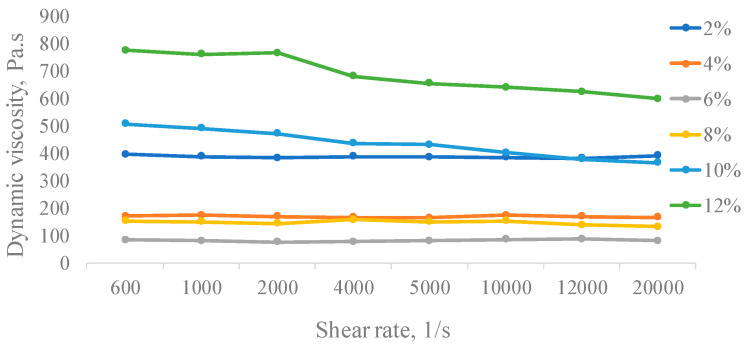
Viscosity of pullulan solutions produced by *A. pullulans* C7 in different concentrations.

The measurement of the dynamic viscosity of the polymer solution obtained by the cultivation of *A. pullulans* C7 showed the dependence on dynamic viscosity at the shear rate at different concentrations ranging from 2% to 12% (*w*/*v*) at 25 °C (Figure 4). The shear rate increased with increasing polymer concentration, thereby demonstrating that viscosity strongly depends on concentration.

At lower concentrations (<8.0%, *w*/*v*), viscosity virtually remained unchanged with increasing shear rate, thus suggesting that the pullulan aqueous solution exhibits Newtonian flow behavior. It is known that Newtonian fluid viscosity is constant no matter the shear rate or applied shear stress experienced by the fluid. However, with increasing concentration to 8% *w*/*v*, the flow behavior was changed to pseudoplastic. Such flow behavior of pullulan can happen because of the separation of exopolysaccharides from each other or the alignment of them with the shear field and thereby a decrease in viscosity up to an approximately constant value [48]. Also, it is known that the viscosity is dependent on the structure and concentration of the polymer, its molecular weight and distribution, the conformation of macromolecules in the solution and its interaction with solvents, the type of intermolecular and intramolecular aggregation, and the flexibility of the chains with temperature. A similar change in flow behavior with increasing concentration was reported for pullulan and other polysaccharides [49,50].

The data obtained allow us to suggest the microbial polymer, pullulan, produced by *A. pullulans* C7 as a potential seed coating binder due to rheological characteristics. It is already known as an excellent film former and is functional for a variety of applications, including for use as an adhesive, binder, and thickener to modify or maintain the texture of food.

It has also been reported that pullulan has considerable mechanical strength and other functional properties such as adhesiveness, film and fiber formability, and enzymatically mediated degradability [51]. High flexibility and a lack of crystallinity provide pullulan with the capacity to form thin layers, electrospun nanofibers, nanoparticles, flexible coatings, stand-alone films, and three-dimensional objects [51,52]. Due to its peculiar characteristics, pullulan is extensively used in different sectors, the three main realms of application pertaining to the pharmaceutical, biomedical, and food fields.

### 3.4. The Use of Various Antifungal Formulations for Seed Treatments in Pot Experiments

Seed coating is a method that involves applying exogenous materials to the surface of seeds to enhance their properties and/or deliver active components (such as plant growth regulators, nutrients, and microbial inoculants). This process can protect seeds from phytopathogens, increase germination rates, improve plant resistance to stress factors, and enhance overall plant growth [37,52,53,54].

**Figure 5:**
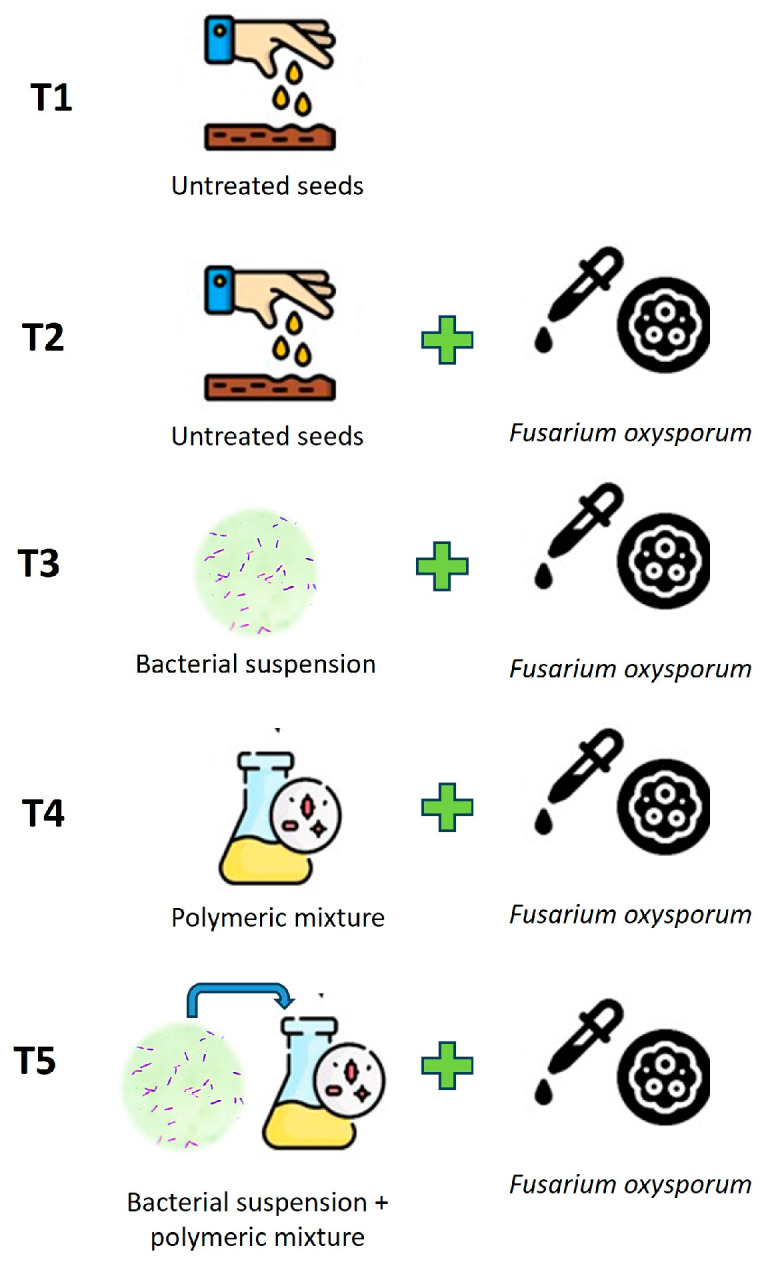
Scheme of pot experiments.

In the next stage of the research, various antifungal formulations for seed coating were developed, and their impact on barley growth under phytopathogenic conditions was assessed (Figure 5).

As active components, a microbial inoculant consisting of a suspension of four compatible strains was used: *Ps. flavescens* D5, *B. aerophilus* A2, *S. proteamaculans* B5, and *Ps. putida* D7. As polymer components, PHA produced by the strain *Ps. flavescens* D5, and pullulan, produced by the yeast strain *A. pullulans* C7, were used. PHA was included in the mixture due to its antifungal properties, as previously identified in earlier studies [15,16].

**Figure 6:**
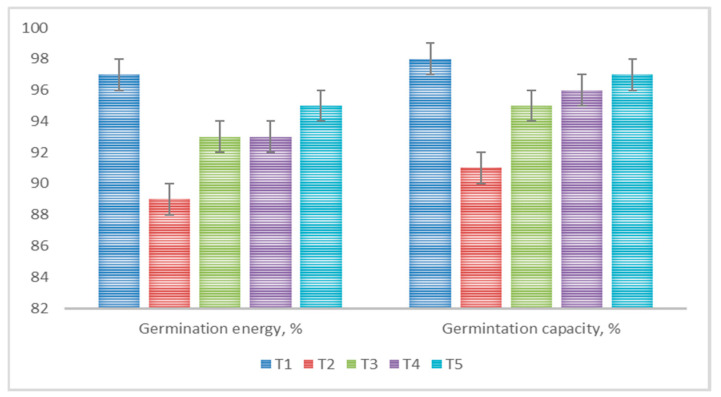
Influence of various pre-sowing treatments on barley seed germination energy and germination capacity.

Uniform seed emergence and early crop development are crucial aspects for achieving high crop yields. Seed coating is an effective method that improves seed-sowing qualities and activates the internal resources of the seed material [52]. In the conducted research, pre-sowing seed treatment, in most cases, enhanced their germination energy and germination capacity. The greatest effect was observed when applying a bacterial suspension in combination with a polymer mixture, where germination energy and germination capacity reached 95% and 97%, respectively (Figure 6).

The pre-sowing treatment of seeds demonstrated a pronounced growth-stimulating effect on barley plants, as evidenced by a significant (*p* < 0.05) increase in morphometric indicators (Table 4).

**Figure 7:**
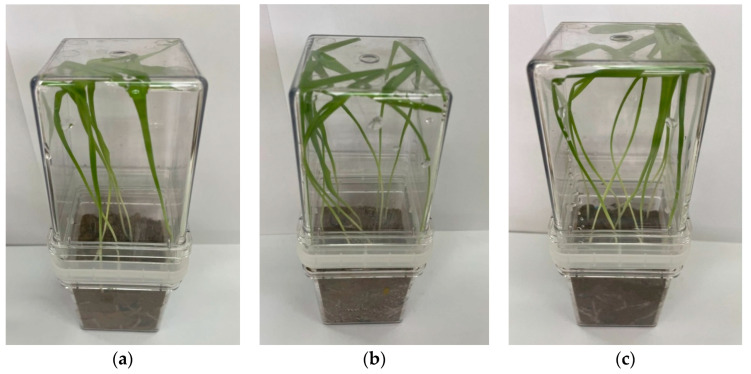
Barley growth under phytopathogenic stress with different seed treatment variants: (**a**) T2—untreated, (**b**) T3—treatment with bacterial suspension, (**c**) T5—seed coating in polymer mixture with bacterial suspension.

The barley’s response varied depending on the type of treatment. Root length is a crucial morphometric indicator as roots are in contact with soil and soil microflora, absorbing water with mineral compounds. The greatest root elongation was observed in variant T5 with the application of a bacterial suspension and a polymer mixture (1.6 times), followed by treatment T3, where root elongation was noted at 1.5 times. Stem length is also a significant characteristic when assessing the plant’s response to different pre-sowing seed treatments. Treated variants showed an increase in stem length by 20–53%. The greatest increase in stem length was observed in variants T3 and T5 (Figure 7). It is shown that the stem mass of treated plants was more than 1.5–1.8 times greater, and root mass was 1.1–1.6 times greater compared to the untreated control (Table 4).

In the conducted research, the observed stimulating effect on the growth parameters of barley can be attributed to several reasons. One of the mechanisms of the positive influence on plants is the ability of the strains included in the composition to produce the phytohormone IAA, which regulates cell division and elongation, their proliferation and differentiation, as well as the development of vascular tissues and apical dominance [55]. Another mechanism for improving morphometric plant parameters under conditions of phytopathogenic stress is the biocontrol properties of strains and the protective role of biopolymers.

The state of the photosynthetic apparatus is an indicator of the physiological condition of plants. One of the primary characteristics of photosynthetic activity is the content of chlorophyll pigments [56]. In previous studies, fluorescence visualization analysis of chlorophyll was applied to assess the condition of the plant photosynthetic system under the influence of biotic [56,57,58] and abiotic [59,60,61] stress. High chlorophyll content may indicate potentially high agricultural productivity [56].

In the present study, under conditions of biotic stress induced by the phytopathogen *F. oxysporum*, the total chlorophyll content in barley leaves decreased by 2.7 times compared to the indicator for plants grown under normal conditions, reaching 1.03 ± 0.03 mg g^−1^ (Table 5). This likely indicates changes in the pigment–protein complexes of light-harvesting antennae and reaction centers of photosystems. Seed treatment had a positive effect on the photosynthetic activity of barley under phytopathogenic stress. This positive effect was to increase the content of chlorophyll *a* in leaves by 1.4–2.1 times, chlorophyll *b* by 2–2.4 times, and the total content of chlorophyll (a + b) by 1.6–2.2 times. The maximum effect was achieved in variant T5 with the application of a bacterial suspension and a polymer mixture (Table 5). The observed differences in pigment content may be associated with the production of certain compounds by the studied bacteria, influencing the biosynthesis and/or degradation processes of chlorophylls, as well as creating more favorable growth conditions for plants under stress.

It is known that stress factors lead to a disruption in the balance between the generation of reactive oxygen species (ROS) and their neutralization. Among the essential mechanisms of plant tolerance mediated by bacteria is the involvement of these microorganisms in detoxifying ROS through the modulation of the natural antioxidant defense systems of plants—both non-enzymatic (proline, ascorbic acid, glutathione, cysteine, flavonoids, carotenoids, and tocopherol) and enzymatic (superoxide dismutase, peroxidase, catalase, ascorbate peroxidase, guaiacol peroxidase, and glutathione reductase), all components of which are in complex functional interaction [62,63].

The increase in proline content is one of the characteristic responses of plants to various types of stress, including biotic stress, providing the first stage of plant adaptation. Proline serves multiple functions, including the regulation of cytosolic acidity, minimization of lipid peroxidation by scavenging free radicals, and stabilization of subcellular components and structures (proteins and membranes) [64]. A higher level of proline in barley leaves was observed when plants were grown in soil with an elevated infectious background compared to untreated plants in sterile soil (Table 5). In the untreated variant under phytopathogenic stress, the proline concentration was 1.7 mg g^−1^, exceeding this indicator in plants grown in favorable conditions by 1.8 times. In treated plants, the proline content was lower. The most noticeable decrease in proline was observed in the variant with simultaneous seed coating in a bacterial suspension and a polymer mixture (Table 5). The obtained results indicate a reduction in the stress experienced by plants due to the pre-sowing seed treatment. Similar to our findings, a reduction in proline levels in various plant species under the influence of microbial treatment has been demonstrated in several studies [65,66].

In the conducted studies, an increase in the activity of antioxidant enzymes was observed when untreated plants were grown under conditions of phytopathogenic stress compared to plants grown in stress-free conditions (Table 6). The obtained data indicate that in response to the action of stress factors, there is an activation of the plant’s defense system.

The pre-sowing seed treatment (T3–T5) led to an increase in catalase activity by 1.3–1.5 times under stress conditions. For ascorbate peroxidase and guaiacol peroxidase, an increase in enzyme activity was observed under phytopathogenic stress in the seed treatment with the bacterial suspension and polymer mixture (T5)—by 2.4 times and 2.7 times, respectively (Table 6). Similarly to the obtained data, previous studies have reported an increase in the activity of antioxidant enzymes in plants when inoculated with bacteria as one of the defense mechanisms of plants when grown under stressful conditions [67,68,69].

Thus, it was shown that when seeds were treated with the T5 composition, plant growth parameters (weight and length of roots) significantly increased compared to the T3 variant with a bacterial suspension. In addition, the use of the T5 composition contributed more to the attenuation of plant stress caused by phytopathogens compared to the use of microorganisms only (Table 5 and Table 6). This indicates that the addition of biopolymers to formulations for seed treatments enhances microbe-induced plant tolerance to phytopathogens.

## 4. Conclusions

As a result of the research, a microbial association of bio-compatible endophytic bacteria has been created, possessing agronomically valuable properties such as the synthesis of the phytohormone IAA, antagonistic activity against *Fusarium oxysporum* and *Fusarium solani*, halotolerance, and PHA production. The study of the rheological properties of polysaccharide solutions showed that pullulan produced by *Aureobasidium pullulans* C7 can be used as seed coating binder at a low concentration of the polymer solution characterized by low viscosity ratio and exhibits Newtonian flow behavior. The effectiveness of various seed coating treatments including biopolymers (PHA and pullulan) and beneficial microorganisms in enhancing the resistance of barley plants to phytopathogens has been demonstrated. The innovative, eco-friendly antifungal seed treatments provide protection for barley against Fusarium diseases, significantly improving seed germination and plant growth in the field. In addition, these polymers will be a new progressive material with the possibility of use in medicine in the form of capsules for prolonged action of drugs, as absorbable suture threads, and dressings. In the form of a film material, the obtained microbial polymers can be used for packaging and storage of food products.

## Figures and Tables

**Table 1:** Plant growth-promoting properties of bacterial strains.

Strains	Properties					
	IAA, μg mL^−1^	Zone of Inhibition of Phytopathogen Growth, cm		Halotolerance		PHA Production, g L^−1^
		*Fusarium solani*	*Fusarium oxysporum*	5% NaCl	15% NaCl	
*Pseudomonas flavescens* D5	45.2 ± 2.1 a	-	3.0 ± 0.1 b	+		2.77 ± 0.07 a
*Bacillus aerophillus* A2	52.4 ± 2.1 b	-	-	+		4.54 ± 0.08 b
*Serratia proteamaculans* B5	62.7 ± 2.1 c	2.6 ± 0.1 b	-	+		
*Bacillus simplex* B9	-	2.1 ± 0.05 a	1.8 ± 0.05 a	+		
*Pseudomonas putida* D7	69.2 ± 3.1 c	-	-	+	+	

Values are given as the mean ± SD. Values represented by the same letter are not significantly different according to the Tukey test (*p* ≤ 0.05).

**Table 2:** Pairwise compatibility among bacterial strains.

Strain	*Pseudomonas flavescens* D5	*Bacillus aerophillus* A2	*Serratia proteamaculans* B5	*Bacillus simplex* B9	*Pseudomonas putida* D7
*Pseudomonas flavescens* D5					
*Bacillus aerophillus* A2	+				
*Serratia proteamaculans* B5	+	+			
*Bacillus simplex* B9	−	−	+		
*Pseudomonas putida* D7	+	+	+	−	

«+»—compatible; «−»—incompatible.

**Table 3:** Production of exopolysaccharides by strains *A. pullulans* C7 and *B. thuringiensis* C8 in presence of glucose.

Strain	The Dry Weight of Cells, g L^−1^ (X)	Production of EPS, g L^−1^ (P)	Utilized Glucose, g L^−1^ (S)	The yield Coefficient for Biomass P/X, %	The yield Coefficient for Substrate P/S, %
*A. pullulans* C7	3.59 ± 0.13	12.53 ± 0.48	17.12 ± 0.81	349.02	73.19
*B. thuringiensis* C8	1.86 ± 0.06	3.97 ± 0.15	10.15 ± 0.61	213.44	39.11

Values are given as the mean ± SD.

**Table 4:** Influence of various pre-sowing seed treatments on growth parameters of barley.

Treatment Variants	Dry Mass of stem, g	Dry Mass of Root, g	Length of Stem, cm	Length of Root, cm
T1	1.2 ± 0.03 d	0.9 ± 0.04 a	22.0 ± 0.9 c	11.5 ± 0.5 b
T2	0.6 ± 0.02 a	0.8 ± 0.03 a	15.0 ± 0.7 a	8.5 ± 0.3 a
T3	1.1 ± 0.04 c	0.9 ± 0.04 a	22.5 ± 0.9 c	12.5 ± 0.2 c
T4	0.9 ± 0.03 b	1.3 ± 0.02 c	20.8 ± 0.8 b	11.0 ± 0.5 b
T5	1.0 ± 0.03 c	1.2 ± 0.03 b	23.8 ± 0.9 c	13.6 ± 0.5 d

Values are given as the mean ± SD. Values represented by the same letter are not significantly different according to the Tukey test (*p* ≤ 0.05).

**Table 5:** Influence of different pre-sowing seed treatments on proline and chlorophyll content in barley.

Treatment Variant	Proline Content, mg g^−1^	Chlorophyll a Content, mg g^−1^	Chlorophyll b Content, mg g^−1^	Total Chlorophyll Content (a + b), mg g^−1^
T1	0.94 ± 0.03 a	1.89 ± 0.07 e	0.94 ± 0.02 e	2.83 ± 0.1 e
T2	1.70 ± 0.07 e	0.69 ± 0.02 a	0.34 ± 0.01 a	1.03 ± 0.04 a
T3	1.4 ± 0.03 d	1.23 ± 0.04 c	0.57 ± 0.02 b	1.8 ± 0.07 b
T4	1.22 ± 0.04 c	0.94 ± 0.03 b	0.68 ± 0.03 c	1.62 ± 0.05 c
T5	1.1 ± 0.03 b	1.42 ± 0.03 d	0.81 ± 0.04 d	2.23 ± 0.07 d

Values are given as the mean ± SD. Values represented by the same letter are not significantly different according to the Tukey test (*p* ≤ 0.05).

**Table 6:** Influence of various pre-sowing seed treatments on the activity of antioxidant enzymes in barley.

Treatment Variant	Catalase, mol min^−1^ mg of Protein ^−1^	Ascorbate Peroxidase, mol min^−1^ mg of Protein^−1^	Guaiacol Peroxidase, mol min^−1^ mg of Protein^−1^
T1	0.12 ± 0.005 a	9.6 ± 0.3 a	4.8 ± 0.2 a
T2	0.23 ± 0.004 b	12.47 ± 0.2 c	7.14 ± 0.3 b
T3	0.34 ± 0.007 d	12.34 ± 0.5 c	6.7 ± 0.3 b
T4	0.35 ± 0.007 d	10.03 ± 0.5 b	6.9 ± 0.3 b
T5	0.29 ± 0.006 c	30.37 ± 0.9 d	19.2 ± 0.7 c

Values are given as the mean ± SD. Values followed by the same letter do not differ according to the Tukey test (*p* ≤ 0.05).

## Data Availability

The data that support the findings of this study are available upon request from the corresponding author.
